# The Colon, the Source of Infectious and Non-Infectious Systemic Diseases*Read before the McLennan County Medical Society, September, 1916.

**Published:** 1916-11

**Authors:** W. F. Cole

**Affiliations:** Waco, Texas


### The Colon, the Source of Infectious and Non-Infectious Systemic Diseases.*

BY DR. W. F. COLE, WACO, TEXAS.

Metchnikoff, the last of that great trio of investigating pathologists, Pasteur, Koch and Metchnikoff, passed away on the 15th of July last, at the age of 71. He who had contended that the normal span of human life should be 150 years instead of 75. He died from an inherited weak heart, which had carried away his forefathers at a much earlier age. He had, as every intelligent person should do, made a complete analysis of himself, phys-

*Read before the McLennan County Medical Society, September, 1916.

ically and mentally, that he might attain the highest efficiency in his life work. Though born and reared on the far off steppes of Russia, by his genius and industry he made himself one of the leading scientists of his time. He discovered the functions of the phagocytes, those wandering white cells in the blood plasma commonly called the leucocytes. He showed how they are the scavengers of the bodily tissues as well as the rebuilders. They engulf and digest the invading bacteria or micro-organisms and waste products, appropriating all the useful elements and carrying away the waste to be excreted from the body. They are endowed with those magical ameboid movements whereby they are enabled to change their form and leak through all the different kinds of bodily tissue to absorb waste tissue, or give themselves as nutriment and disappear. Metchnikoff demonstrated how they are, literally, the slaves of the bodily tissues, being defenders, nourishers and scavengers. When a wound occurs at any point the phagocytes swarm to that point to rebuild the wounded tissues and to defend against infection. If successful they localize the infection to the point of the wound, which accounts for the inflammation at that point. For his discoveries he was awarded the Nobel prize in 1898. For his more recent discoveries concerning the cause of immunization he was awarded the Albert medal not long before his death. He is best known, however, for his theories concerning the cause of old age, which he attributed to micro-organisms developed in the intestinal tract. Basing his theories on the functions of the phagocytes, he believed that malignant micro-organisms might be combated with benign organisms. He studied the tables of longevity of the whole world, and found that man attains his greatest age in the Balkan peninsula, where one of the staple articles of diet is a fermented milk having its own specific bacteria. He believed that this robust and benign bacteria performed a phagocytic function in the intestinal tract, destroying the malignant types, or at least crowding them out, as some forms of vegetation crowd out other forms. This specific fermenting bacteria is extensively used to produce the so-called Bulgarian buttermilk. The deductions of Metchnikoff have been pretty generally accepted by the medical profession, and his teachings are generally incorporated in all books on hygiene. I look upon his life work as marking an era in medical science. From my own observations and experience I have found his teaching to be scientifically exact. Primeval man knew his enemies to be the cave bear, the wolf and other carnivora; he knew nothing of those myriads of unseen microorganisms that are the bane of modern man, and which are far deadlier than all the visible enemies with which the human race has to contend. The medical profession is as old as the human race. We cannot conceive a time when man did not require aid to dress his wounds and minister to his ailments, yet it has been one of the most conservative and dogmatic of all the professions. I believe that medical science has made more progress during the lifetime of those whom I address than it did during the whole history of the human race. Hippocrates was contemporary with Phidias, whose works in architecture and sculpture have rarely if ever been equaled and never excelled; yet Hippocrates knew scarcely anything of scientific value in medicine; though his theories were the basis of all medical teaching for more than two thousand years, though so irrational and absurd. He was the author of the celebrated theory of the humors. He taught that the body contains four humors, blood, phlegm, yellow bile and green bile; a right proportion and mixture of which constitutes health, while an improper proportion and mixture disease.

Even as late as the fifteenth century, when Florence was the glory of the renaissant period, and the home of such men as Michael Angelo, Raphael, Petruchio, Leonardo da Vinci and Machiavelli, the science, if it could be so called, of medicine was at a very low ebb. When Lorenzo de Medici became ill at the age of forty-two, the most eminent physicians in all Italy were called into consultation. After mature deliberation they stated that there was only one preparation in the pharmacopeia that might possibly effect a cure. This, they stated, was a potion containing powdered emeralds, topazes, rubies and other precious stones. When Lorenzo, the Magnificent, was given this potion, he was promptly gathered unto his fathers, the same as if he had been given powdered glass. History does not so record the fact, but it should state that Lorenzo was slain by his eminent physicians. It is a blessing to the world that medicine is becoming, as it should be, an exact science, rather than a mass of dogmatic theories.

The quack is still among us, but, thanks to the increasing intelligence of the masses, he is passing away to join his brethren, the hoodoo and the witch doctors. It has been said, and truthfully in the past, that medicine was not an exact science. This can no longer be truthfully said, thanks to the labors of such men as Harvey, Pasteur, Lyster, Koch, Metchnikoff, and a host of others who are following in their footsteps. Materia medica has many exceptions because of the idiosyncrasies of individuals, but anatomy, physiology and pathology may be considered exact sciences. The mysteries of pathology have been explained by those noble investigators mentioned, so that today this branch of medicine may be considered an exact science, even as astronomy, and can be demonstrated by the natural law of cause and effect. For more than a century before 1845 astronomers had observed the eccentricity in the orbit of Uranus, but no one had advanced a rational cause for it. Then occurred, simultaneously, those wonderful calculations made by Adams, the Englishman, and Leverrier, the Frenchman. Both attributed the eccentricity in the radius vector of Uranus to the attraction of an unknown planet. Leverrier sent his calculations to the observatory at Berlin, with instructions for pointing the telescope. The planet Neptune was readily found, as indicated by Leverrier. Pathology, like astronomy, is controlled by nature's laws of cause and effect, which change not; therefore, we can look upon pathology as an exact science. Scientific investigations have proven, absolutely, that every infectious disease has its own specific causal micro-organism, whether bacterium or protozoa. It is not guesswork any longer with the diagnostician. When the symptoms of disease resemble those of cholera he looks for the comma bacillus of Koch, and if he finds it, he is as sure of the cause of the disease as he would be that water will boil at 212 degrees at sea level. The specific causal micro-organisms of all diseases have not been discovered, but we are as sure that they exist as Adams and Leverrier were that an unknown planet caused the eccentricity in the orbit of the planet Uranus.

For this reason I have contended that pellagra, a typical infectious epidemic disease, is caused by some specific micro-organism, either bacterium or protozoa. I have been greatly surprised that so many physicians have been stampeded into the adoption of that absurd and irrational theory that an unbalanced diet is the cause of the great epidemic which has raged in the South during the last few years. That proper nourishing food enables the bodily tissues to resist the invasion of micro-organism is axiomatic. In the *Southern Medical Journal* for May, 1916, I reported the cure of a case of pellagra in which the patient was looked upon as hopeless. This patient is here this evening for your inspection. He is, as you perceive, free from any symptoms of pellagra, and has been so since November, 1915. This patient was in the last stage of emaciation, and his neck, arms, legs and feet were almost one mass of scabs where he had scratched himself. He cringed when anyone approached and his eyes glittered with maniacal fear. His bowels moved many times daily, and the stools were mixed with blood and mucus, and literally loaded with colon bacilli. I gave him calomel and soda each three grains with santonin one grain at bed time, followed with oil in the morning. This was repeated the following evening. The blood ceased on the second day and did not reappear. Broken doses of calomel and soda were given every few days, followed with oil till he recovered entirely. This case was as typical as any that ever was. His father died of pellagra and the mother was ill with it for several months. I have received many letters from all parts of the South, from physicians, stating that they had used the treatment with the very best results. At first, I suspected that the causal germ might be a protozoa, but now I suspect that it may belong to the family of the colon bacilli, which has not been differentiated from the many varieties of the colon-typhoid types. My conclusions are that the micro-organism develops in the intestinal tract, most likely in the colon, and that the nervous and skin symptoms are due to toxins developed there. The sympathetic nervous system is profoundly affected by the toxins, as in the case of scarlet fever, measles and other exanthems. Since the toxins originate in the intestinal tract, particularly in the colon, the rational treatment is to sweep it out with a powerful antiseptic. For this purpose I know of nothing better than calomel and oil. As for the oil, I have gotten good results from the use of the refined petroleum, which I used with castor oil in this case before you. The use of this oil is rational, since it forms a menstruum in which germs do not flourish.

I desire to call your attention briefly to some diseases that are usually classed as non-infectious, such as gout, rheumatism and piles. Those diseases, like pellagra, I attribute to colon bacilli and putrefactive conditions in the colon. I cannot explain this case better than to give you the history of a case which I am able to exhibit to you this evening. This gentleman is nearly sixty years of age, and had always been exceedingly healthy up to one year since, when he began to take on flesh very rapidly. His weight increased from the normal of thirty years of 176 to 205 pounds. Though his bowels moved regularly every day and his urine was normal, he began to have periodical headaches every few days, and he was simultaneously attacked with rheumatism in the hands and gout in the right foot, which was so severe that he was disabled entirely for a time. He had suffered slightly for several years at times from protruding piles, but at no time was he seriously discommoded. The piles now became exceedingly painful, and in a short time he was subject to bleeding, which became so intense that he was seriously weakened from loss of blood. This patient was under the care of one of the best physicians in this city, and took the routine treatment faithfully, including diet, but with little or no improvement. When a man decides to take his own case in hand he is said to be like the lawyer who is said to have a fool for a client when he becomes his own attorney. However the case may be, when an individual takes counsel with himself he is pretty sure to put theories away in the background and rely on common sense reasoning, which, going hand in hand with science, is the foundation of success in every calling. I should have mentioned that this patient had taken on the typical aldermanic abdomen, in other Anglo-Saxon language, he had become pot-bellied. He suffered at times from acute and painful inflammations of the transverse and descending colon, and there was some tenderness over this region most all the time. On examination of the feces, I found it loaded with colon bacilli and exceedingly acid in reaction, and quite offensive in odor. I decided that his gout and rheumatism was due to toxaemia from the colon, the effects of putrefactive conditions brought about by colon bacilli, which also accounted for the excessive acidity of the feces. I concluded that the excessive acidity of the feces in the rectum was most likely the cause of the piles. I cleansed the lower bowel with an enema of castile soapsuds and injected two ounces of olive oil containing two grains of calomel. During two days I gave broken doses of calomel, preceded by paraffine oil. My intention was to cleanse the whole intestinal tract and get it as nearly aseptic as possible. This patient has had no piles from that day to this; neither has he had the least symptom of either rheumatism or gout. His weight has been reduced to the old normal of 178.

I beg to say to you, gentlemen, that I am the patient. I am sixty years old, but am, if possible, more healthy now, physically and mentally, than I was at forty. I might mention that I am simple in my diet, that I adapt my food to my necessities and not to my appetite. I eat little meat, and when I feel under tone physically or mentally I fast. I go to the stool twice every day instead of only once as in former times.

In conclusion, I beg to repeat that I am in complete accord with Metchnikoff in his conclusions that the majority of human ailments that produce premature old age and death are due to putrefactive conditions in the colon. The cure for this is simple.

I should mention that my girth measure has been reduced four inches. When I see an individual full blooded and pot-bellied I know that he is as full of toxins as a lemon with juice, and that he is going with express speed to the grave, from arteriosclerosis or kidney disease, or some other functional trouble. The great Russian is no longer among the living, but his teachings remain, a perpetual heritage of the human race.

---

Little Willie became slightly indisposed, and when the family doctor was called he prescribed some medicine in powder form.

"Come, Willie," said the fond mother, preparing one of the powders as soon as the medicine arrived from the drug store, "you must take this right away, so that you will be well."

"No, I don't want to take it," whined Willie, backing away from the dose. "I don't need no medicine."

"Why, Willie," pleaded mother, gently drawing the boy toward her; "you never heard me complain about a little powder, did you?"

"No, an' neither would I," was the startling rejoinder of Willie, "if I could just put it on my face like you do; but I have to swallow it."—*Philadelphia Telegraph.*

---

After a hurried rush through the night the doctor found his patient in a very bad way.

"My dear sir," he said slowly, "I have been attending you for nine weeks and have done my best, but I'm afraid that your end is near. Have you any last wish to express?" The patient drew a long breath.

"Yes, doctor," he replied, in a faint voice. "I wish I had had another doctor!"—*Temple Telegram.*

---

Customer: "Are you sure this article will cure my rheumatism?"

Clerk: "Oh, yes; all the doctors refused to recommend it." —*Cushing Enterprise.*

---

Brown—I should think doctors would be even more tyrannical and autocratic than they are.

Smith—Why so?

Brown—Because all their dealings are with people who are in no condition to fight back.—*Life.*

